# Preliminary Findings of a Randomized Trial of Non-Pharmaceutical Interventions to Prevent Influenza Transmission in Households

**DOI:** 10.1371/journal.pone.0002101

**Published:** 2008-05-07

**Authors:** Benjamin J. Cowling, Rita O. P. Fung, Calvin K. Y. Cheng, Vicky J. Fang, Kwok Hung Chan, Wing Hong Seto, Raymond Yung, Billy Chiu, Paco Lee, Timothy M. Uyeki, Peter M. Houck, J. S. Malik Peiris, Gabriel M. Leung

**Affiliations:** 1 Department of Community Medicine and School of Public Health, Li Ka Shing Faculty of Medicine, The University of Hong Kong, Hong Kong, China; 2 Department of Microbiology, The University of Hong Kong, Hong Kong, China; 3 Queen Mary Hospital, Hospital Authority, Hong Kong, China; 4 Centre for Health Protection, Department of Health, Government of the Hong Kong SAR, China; 5 Hong Kong Sanatorium and Hospital, Hong Kong, China; 6 St Paul's Hospital, Hong Kong, China; 7 Influenza Division, Centers for Disease Control and Prevention, Atlanta, Georgia, United States of America; 8 Division of Global Migration and Quarantine, National Center for Preparedness, Detection and Control of Infectious Diseases, Centers for Disease Control and Prevention, Seattle, Washington, United States of America; Fred Hutchinson Cancer Research Center, United States of America

## Abstract

**Background:**

There are sparse data on whether non-pharmaceutical interventions can reduce the spread of influenza. We implemented a study of the feasibility and efficacy of face masks and hand hygiene to reduce influenza transmission among Hong Kong household members.

**Methodology/Principal Findings:**

We conducted a cluster randomized controlled trial of households (composed of at least 3 members) where an index subject presented with influenza-like-illness of <48 hours duration. After influenza was confirmed in an index case by the QuickVue Influenza A+B rapid test, the household of the index subject was randomized to 1) control or 2) surgical face masks or 3) hand hygiene. Households were visited within 36 hours, and 3, 6 and 9 days later. Nose and throat swabs were collected from index subjects and all household contacts at each home visit and tested by viral culture. The primary outcome measure was laboratory culture confirmed influenza in a household contact; the secondary outcome was clinically diagnosed influenza (by self-reported symptoms). We randomized 198 households and completed follow up home visits in 128; the index cases in 122 of those households had laboratory-confirmed influenza. There were 21 household contacts with laboratory confirmed influenza corresponding to a secondary attack ratio of 6%. Clinical secondary attack ratios varied from 5% to 18% depending on case definitions. The laboratory-based or clinical secondary attack ratios did not significantly differ across the intervention arms. Adherence to interventions was variable.

**Conclusions/Significance:**

The secondary attack ratios were lower than anticipated, and lower than reported in other countries, perhaps due to differing patterns of susceptibility, lack of significant antigenic drift in circulating influenza virus strains recently, and/or issues related to the symptomatic recruitment design. Lessons learnt from this pilot have informed changes for the main study in 2008.

**Trial Registration:**

ClinicalTrials.gov NCT00425893

HKClinicalTrials.com HKCTR-365

## Introduction

The specter of an influenza pandemic continues to threaten, with annual outbreaks of highly-pathogenic H5N1 in birds [1] and continued sporadic human H5N1 cases and clusters [2] with some reports that suggested limited, non sustained human-to-human transmission of H5N1 viruses [3], [4]. If a pandemic virus strain were to emerge, pre-pandemic vaccines would be available to some populations although of unknown efficacy, but development and distribution of initial doses of influenza vaccine specifically made against the pandemic strain would not be available for at least 4–6 months [5]. Influenza antiviral medications would likely be in short supply in many regions, particularly in developing countries, and might have modest effectiveness against the pandemic strain, because of the emergence of antiviral resistance or other reasons [6]. Furthermore, few of these pharmaceutical measures can be applied at pandemic scale. Only non-pharmaceutical interventions [7]–[12] including use of face masks, improved hand hygiene, cough etiquette, social distancing measures, and travel restrictions would be available to the majority of the world's population. Interpandemic influenza is associated with thousands of deaths every year in Hong Kong [13] and likely hundreds of thousands worldwide every year [14], [15], therefore simple personal protective measures could be beneficial during annual epidemics if found to be effective in reducing transmission, and as an adjunct to influenza vaccination.

We implemented a prospective cluster-randomized trial [16] to test whether two such non-pharmaceutical interventions can reduce transmission of interpandemic influenza in households.

## Methods

The protocol for this trial and supporting CONSORT checklist are available as supporting information; see Protocol S1 and Checklist S1.

### Recruitment and follow-up of participants

From 30 first-contact outpatient clinics in both the private and public sectors across Hong Kong, we enrolled 944 Hong Kong residents aged at least 2 years, reporting at least two symptoms of influenza-like-illness (ILI) (such as fever ≥38°C, cough, sore throat, coryza, headache, malaise, chills, fatigue, etc.), and living in a household with at least two other individuals none of whom had reported ILI symptoms in the preceding 14 days. These index subjects provided nasal and throat swab (NTS) specimens which were combined and tested with the QuickVue Influenza A+B rapid diagnostic test (Quidel Corp, San Diego, CA) and those subjects with a positive result for influenza A or B were randomized and further followed up. For participants enrolled after June 1, 2007, those index subjects with a negative QuickVue result but a fever ≥38°C were also randomized and further followed up. Data on clinical signs and symptoms were collected for all subjects, and an additional NTS was collected for later confirmation of influenza infection by viral culture.

Following randomization a home visit was scheduled (to take place within 36 hours) to implement the intervention, collect baseline demographic data and NTS from all household members aged ≥2 years, and to provide and describe proper use of a free tympanic thermometer and the daily symptom record sheets. During the 9 days following the initial home visit, all household members were asked to keep symptom diaries, and three further home visits were scheduled at 3, 6 and 9 days after the baseline household visit to monitor adherence to interventions and to collect further NTS from all household members aged ≥2 years. At the final (day 9) home visit, the study nurse collected the symptom diaries and evaluated adherence to interventions by interview and by counting the number of surgical masks remaining or weighing the amount of soap and alcohol left in bottles and dispensers.

### Ethics

All subjects aged 18 years and older gave written informed consent. Proxy written consent from parents or legal guardians was obtained for subjects aged 17 years and younger, with additional written assent from those aged 8 to 17 years. The study protocol was approved by the Institutional Review Board of the University of Hong Kong/Hospital Authority Hong Kong West Cluster and was conducted in compliance with the Declaration of Helsinki [17].

### Interventions

Our study compared three interventions. In the control arm, households received education about the importance of a healthy diet and lifestyle, both in terms of illness prevention (for household contacts) and symptom alleviation (for the index). Households in the face mask arm received the control intervention plus education about the potential efficacy of masks in reducing disease spread to household contacts if all parties wear masks, distribution of a box of 50 surgical masks (Tecnol – The Lite One, Kimberly Clark, Roswell, GA) for each household member (or a box of 75 paediatric masks for children aged 3–7 years), and demonstration of proper face-mask wearing and hygienic disposal. Index subjects and all household contacts were taught to wear masks as often as possible at home (except when eating or sleeping) and also when the index was with the household members outside of the household. Households in the hand hygiene group received the control intervention plus education about the potential efficacy of proper hand hygiene in reducing transmission, distribution of an automatic alcohol hand sanitizer (WHO recommended formulation II, liquid content with 75% isopropyl alcohol, Vickmans Labs Ltd., Hong Kong), liquid hand soap (Avalon organics glycerin hand soap, Petaluma, CA), individual small (125 ml) bottles of alcohol hand gel (Gellygen gel with 70% ethyl alcohol, Brymore SA, Italy), and demonstration of proper hand washing and hand antisepsis [18]. All household members including the index subject were taught to use the liquid soap in place of their regular soap after every washroom visit and in general when their hands were soiled or after sneezing or coughing, while they should use the alcohol hand sanitizer or hand rub when first returning home and immediately after touching any potentially contaminated surfaces. At the final home visit, households were reimbursed for their participation time with a supermarket voucher worth approximately US$20.

### Objectives

The overall objective of the study was to quantify the efficacy of face masks and/or hand hygiene in reducing transmission of influenza to household contacts at the individual level. Specific objectives of this pilot study were to confirm the feasibility of the study design including the practicability of patient recruitment, randomization and follow-up, the appropriateness of the estimated sample size for a subsequent larger trial in terms of characteristics of local circulating influenza viruses and potential effect sizes, the applicability of the interventions and individual adherence with the interventions.

### Outcomes

The primary outcome measure was the secondary attack ratio (SAR) at the individual level i.e. the proportion of household contacts of an index case who subsequently became ill with influenza. We evaluated the SAR using a laboratory definition (at least one follow-up NTS positive for influenza by viral culture or PCR) as the primary analysis, and three different clinical definitions of influenza as secondary analyses. The first definition of clinical influenza was fever ≥38°C or at least two of the following symptoms: headache, coryza, sore throat, aches or pains in muscles or joints, cough, or fatigue. The second definition was at least two of the following signs and symptoms: fever ≥37.8°C, cough, headache, sore throat, aches or pains in muscles or joints [19]. The third definition was the standard WHO/CDC influenza-like illness definition: fever ≥37.8°C plus cough or sore throat [20]. A secondary outcome measure was the secondary attack rate (SAR) at the household (cluster) level i.e. the proportion of households with one or more secondary case.

### Sample size

We estimated that we would require 51 households (average size 3.8) in the control arm to allow determination of a secondary attack ratio of approximately 24% [21] to within +/−7%. Allowing for potential dropout, we therefore planned to recruit at least 60 households in the control arm, and a further 25–30 households to each of the face mask and hand hygiene arms to evaluate the feasibility of the interventions and allow a preliminary albeit imprecise estimate of efficacy. This pilot study was not powered to detect small or moderate efficacies of the interventions with statistical significance. We did not specify any early stopping rules or interim analyses.

### Randomization

Randomization lists were prepared by a biostatistician (B.J.C.). Eligible study participants were randomly allocated to three groups. The first 100 households were randomized in the ratio 2∶1∶1 and subsequent households were randomized in the ratio 8∶1∶1 using a random number generator (R software). The rationale for changing the randomization ratio was to allow us to gather maximum information about the natural characteristics of influenza transmission in households in the absence of control measures, after evaluating the feasibility of each of the interventions in at least 25 households. Interventions were assigned to households by the study manager (R.O.P.F.) based on the randomization sequence. The allocation to specific intervention arms was concealed to recruiting doctors/clinics throughout.

### Blinding

Participants and those administering interventions were not blinded to the interventions, but participants were not informed of the specific nature of the other interventions applied to other participating households.

### Laboratory methods

Nasal swabs were collected by inserting and rotating a sterile swab (Collection swab; EUROTUBO, Madrid, Spain) into the anterior nares. Throat swabs were collected by rubbing a second sterile swab against the tonsillar fossa. Both swabs were snapped off into a tube containing viral transport medium (5% bovine serum albumin in Earle's balanced salt solution with antibiotic). At recruitment, additional nose and throat swabs were collected using sterile foam swabs and then combined and tested by the QuickVue Influenza A+B rapid diagnostic test.

Specimens collected from index subjects at recruitment were stored in a 2–8°C refrigerator (overnight, if required). Specimens collected during home visits were stored in a cool box with at least two icepacks immediately after collection. Before the end of the day of a home visit, study nurses took samples to the nearest collection point for storage in a 2–8°C refrigerator (overnight, if required) or directly to the central testing laboratory. Samples stored at 2–8°C were delivered to the central testing laboratory by courier in cool boxes en route. Samples were eluted and cryopreserved at −70°C immediately after receipt.

All clinical specimens were cultured on Madin-Darby canine kidney cells with exogenous trypsin (2 ug/ml) added. In households which were successfully followed up with home visits, the clinical specimens collected from index subjects at the recruiting clinic and during the first home visit were additionally tested by reverse transcription polymerase chain reaction (RT-PCR) for influenza A and B viruses if both specimens were negative by viral culture. For household contacts who reported symptoms during the follow-up but whose corresponding clinical specimens (collected within +/−2 days of self-reported fever or other respiratory symptoms) were negative by viral culture, those specimens were additionally tested for influenza A and B by RT-PCR. Additional technical details of the laboratory procedures employed in viral culture and RT-PCR testing are given in Text S1.

### Statistical methods

To evaluate the SAR and to compare between groups we used exact binomial 95% confidence intervals, and χ^2^ tests and multivariable logistic regression models adjusting for potential within-household correlation [22], [23], with a 5% type I error rate. We estimated the intra-cluster correlation coefficient from the mean squared errors in the SAR between and within households [22]. All analyses were by intention-to-treat. We evaluated the three definitions of clinical influenza described above using receiver operating characteristic (ROC) analysis to determine the clinical definition that corresponds most closely to the laboratory outcome measure [24]. All analyses were conducted in R version 2.4.1 [25].

## Results

**Figure 1:**
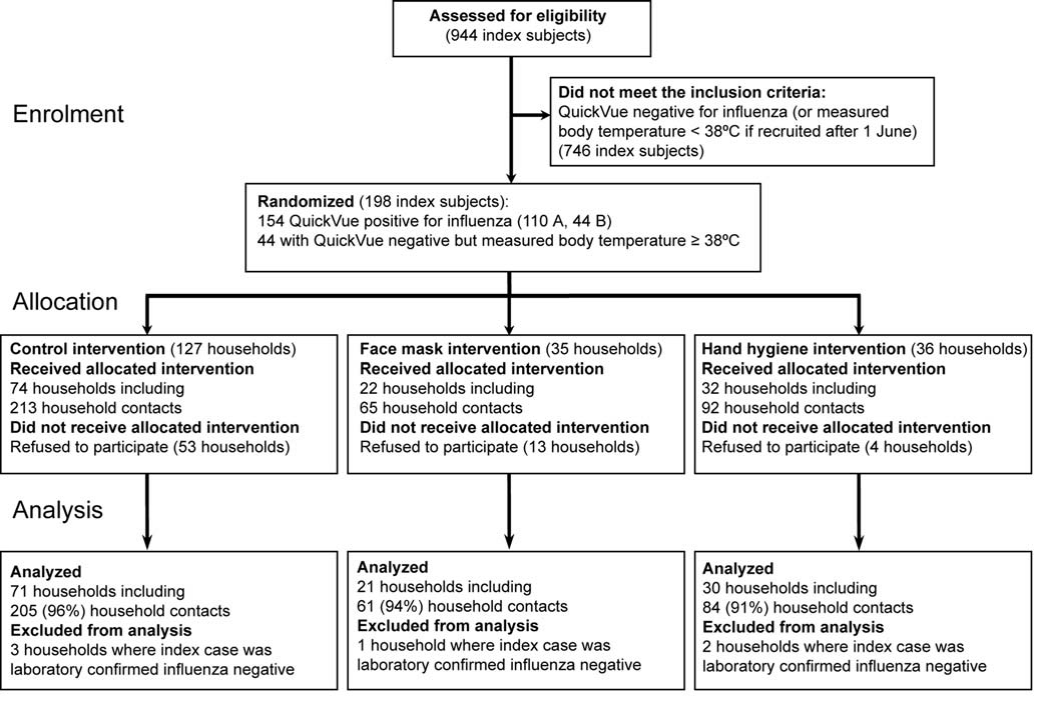
Flow of subjects through the study.

Nine hundred and forty-four subjects were initially recruited to the study between February 24 and September 14, 2007. Figure 1 shows the progress of subjects and household contacts through the study. Overall, and in each intervention arm, the median household size was 4. Both the recruitment rate of subjects and the percentage of positive rapid influenza test results among recruited subjects increased in line with other measures of influenza activity including sentinel outpatient visits and laboratory isolations in mainly inpatient specimens during the periods of peak influenza activity in February and June (Figure S1). Of the 944 recruited subjects, 198 met the criteria for randomization and further follow-up. In a protocol deviation we randomized 9 subjects who had symptoms for (slightly) more than 48 hours; these 9 subjects were retained in the analyses.

### Baseline data

Characteristics of the 198 subjects are shown in Table 1 according to intervention arm. In general the groups were well-matched. After randomization 70 (35%) of the households declined any home visits or could not be contacted after numerous repeated attempts. Proportionally more of these dropouts were in households where the index was a young adult, whereas there were few dropouts when the index subject was a child. Dropout was higher in households of index subjects who had a negative result on the rapid influenza test (25/44, 57%) compared to those who had a positive result (45/154, 29%).

**Table 1:** Characteristics of 198 randomized index subjects by intervention arm; the 128 index subjects successfully followed with home visits and their 370 household contacts.

	Control				Face mask				Hand hygiene			
*Index subjects*	Randomized (n = 127)		Followed up (n = 74)		Randomized (n = 35)		Followed up (n = 22)		Randomized (n = 36)		Followed up (n = 32)	
Age group (%)												
2–15 years	48	(38%)	33	(45%)	12	(34%)	9	(41%)	13	(36%)	12	(38%)
16–30 years	23	(18%)	10	(14%)	7	(20%)	3	(14%)	7	(19%)	6	(19%)
31–50 years	32	(25%)	17	(23%)	11	(31%)	6	(27%)	10	(28%)	10	(31%)
50+ years	24	(19%)	14	(19%)	5	(14%)	4	(18%)	6	(17%)	4	(12%)
No. (%) men	60	(47%)	32	(43%)	16	(46%)	12	(55%)	14	(39%)	12	(38%)
Symptoms (%)												
Cough	99	(78%)	62	(84%)	24	(69%)	13	(59%)	33	(92%)	29	(91%)
Runny nose	98	(77%)	61	(82%)	28	(80%)	16	(73%)	28	(78%)	26	(81%)
Fatigue / tiredness	96	(76%)	56	(76%)	26	(74%)	16	(73%)	29	(81%)	25	(78%)
Fever (body temperature≥38°C)	94	(74%)	54	(73%)	25	(71%)	17	(77%)	29	(81%)	27	(84%)
Headache	80	(63%)	40	(54%)	29	(83%)	18	(82%)	22	(61%)	19	(59%)
Sore throat	69	(54%)	37	(50%)	23	(66%)	13	(59%)	22	(61%)	19	(59%)
Aches / pains in muscles or joints	62	(49%)	34	(46%)	18	(51%)	9	(41%)	18	(50%)	16	(50%)
Onset to randomization interval (%)												
0–24 hours	86	(68%)	48	(65%)	21	(60%)	14	(64%)	25	(69%)	22	(69%)
24–48 hours	35	(28%)	22	(30%)	12	(34%)	8	(36%)	7	(19%)	7	(22%)
48+ hours	5	(4%)	4	(5%)	1	(3%)	0	(0%)	3	(8%)	3	(9%)
Household contacts			(n = 213)				(n = 65)				(n = 92)	
Age group (%)												
0–15 years	–	–	32	(15%)	–	–	11	(17%)	–	–	14	(15%)
16–30 years	–	–	43	(20%)	–	–	13	(20%)	–	–	17	(18%)
31–50 years	–	–	92	(43%)	–	–	28	(43%)	–	–	35	(38%)
50+ years	–	–	43	(20%)	–	–	12	(18%)	–	–	25	(27%)
No. (%) men	–	–	83	(39%)	–	–	26	(40%)	–	–	37	(40%)
Influenza vaccination in the previous 12 months	–	–	29	(14%)	–	–	3	(1%)	–	–	12	(6%)

**Figure 2:**
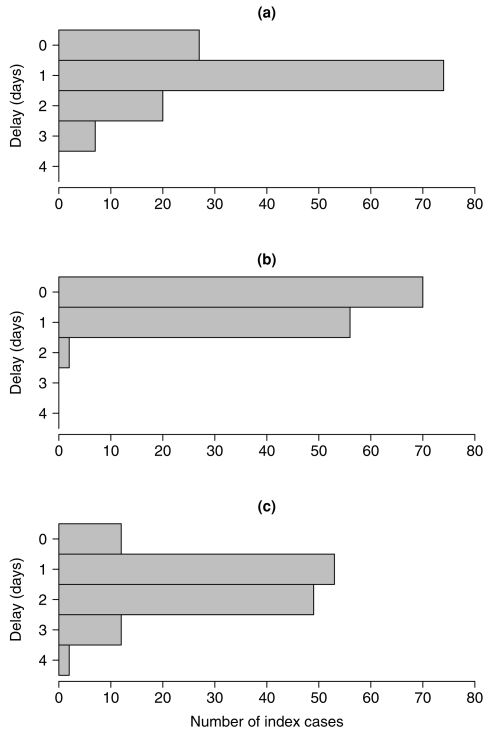
Delays between index case symptom onset, randomization, and intervention in 128 households. Time intervals a) from symptom onset in the index subject to randomization; b) from randomization to application of the intervention; c) from symptom onset to application of the intervention.

We implemented the interventions in the remaining 128 households, and 127 (99%) were successfully followed for all four home visits; one household completed three home visits. (Table 1) The median household sizes were 4 in all intervention arms. We were typically able to apply the intervention within 1–2 days of symptom onset in the index case (Figure 2). Delays between symptom onset and intervention did not significantly differ between study arms (data not shown).

### Numbers analyzed

Influenza could not be confirmed by viral culture or RT-PCR in the index subjects in 6 of the 128 households; therefore we only retained 122 households for analysis of crude SARs. Five household contacts had missing data on age, and these were further excluded for the multivariable regression analyses.

### Main outcomes

The overall laboratory-confirmed SAR was 6.0% (95% confidence interval 3.8%–9.0%) while the clinical SARs were 18%, 11% and 5% according to the three alternative definitions, respectively, with little difference between intervention arms (Table 2). The within-household correlation was 0.18 for the laboratory-confirmed SAR and varied from −0.05 to 0.01 for the various clinical definitions of influenza; chi-squared tests for differences in SARs between intervention arms were adjusted for these correlations (Table 2). The SARs were similar when stratified by the delay between onset of symptoms in the index case and application of the intervention (Table 2). Overall, 17/122 (14%) households had one or more laboratory-confirmed secondary case, while 44 (36%), 29 (24%) and 14 (11%) had one or more clinical secondary cases according to the three definitions above, respectively. SARs were similar when stratifying by influenza A or B infection in the index case (data not shown).

**Table 2:** Secondary attack ratios of laboratory-confirmed influenza and clinical influenza in the contacts of 122 analyzed households, by intervention arm.

Interval between symptom onset and intervention		Secondary attack ratio (95% CI*)						*p*-value†
		Control		Face mask		Hand hygiene		
		(n = 205)		(n = 61)		(n = 84)		
Any	Laboratory confirmed influenza	0.06	(0.03, 0.10)	0.07	(0.02, 0.16)	0.06	(0.02, 0.13)	0.99
	Clinical influenza definition 1‡	0.18	(0.13, 0.24)	0.18	(0.09, 0.30)	0.18	(0.10, 0.28)	1.00
	Clinical influenza definition 2‡	0.11	(0.07, 0.16)	0.10	(0.04, 0.20)	0.11	(0.05, 0.19)	0.97
	Clinical influenza definition 3‡	0.04	(0.02, 0.08)	0.08	(0.03, 0.18)	0.04	(0.01, 0.10)	0.52
		(n = 110)		(n = 32)		(n = 41)		
≤36 hours	Laboratory confirmed influenza	0.06	(0.03, 0.13)	0.12	(0.04, 0.29)	0.10	(0.03, 0.23)	0.69
	Clinical influenza definition 1‡	0.17	(0.11, 0.26)	0.25	(0.11, 0.43)	0.17	(0.07, 0.32)	0.76
	Clinical influenza definition 2‡	0.11	(0.06, 0.18)	0.09	(0.02, 0.25)	0.10	(0.03, 0.23)	0.98
	Clinical influenza definition 3‡	0.04	(0.01, 0.09)	0.09	(0.02, 0.25)	0.05	(0.01, 0.17)	0.44
		(n = 95)		(n = 29)		(n = 43)		
>36 hours	Laboratory confirmed influenza	0.05	(0.02, 0.12)	0.00	(0.00, 0.12)	0.01	(0.00, 0.12)	0.30
	Clinical influenza definition 1‡	0.19	(0.12, 0.28)	0.10	(0.02, 0.27)	0.19	(0.08, 0.33)	0.71
	Clinical influenza definition 2‡	0.12	(0.06, 0.20)	0.10	(0.02, 0.27)	0.12	(0.04, 0.25)	0.99
	Clinical influenza definition 3‡	0.05	(0.02, 0.12)	0.07	(0.01, 0.23)	0.02	(0.00, 0.12)	0.79

* Confidence intervals were calculated by the exact binomial method, not accounting for within-household correlation, and the resulting intervals may therefore slightly underestimate the uncertainty about the SARs.

† By Pearson chi-square test adjusted for within-household correlation.

‡ Clinical influenza definition 1 is fever≥38°C or at least 2 of headache, runny nose, sore throat, aches or pains in muscles or joints, cough, or fatigue. Clinical influenza definition 2 is at least 2 of fever≥37.8°C, cough, headache, sore throat, aches or pains in muscles or joints. Clinical influenza definition 3 is the standard CDC classification of fever≥37.8°C plus cough or sore throat.

Table 3 shows the odds ratios of secondary infection in a household contact by intervention arm, adjusted for age, sex, influenza vaccination history and the age and sex of the corresponding index subject. Results were similar when stratified by the delay between symptom onset and application of the intervention (data not shown).

**Table 3:** Factors affecting the laboratory-confirmed influenza and clinical influenza secondary attack ratios in the 350 household contacts.

	n	Laboratory-confirmed influenza		Clinical influenza*					
				Definition 1		Definition 2		Definition 3	
		OR†	95% CI for OR	OR†	95% CI for OR	OR†	95% CI for OR	OR†	95% CI for OR
Control group	202	1.00		1.00		1.00		1.00	
Face mask group	60	1.16	(0.31, 4.34)	0.88	(0.34, 2.27)	0.87	(0.30, 2.51)	2.00	(0.57, 7.02)
Hand hygiene group	83	1.07	(0.29, 4.00)	0.86	(0.39, 1.91)	0.88	(0.36, 2.14)	0.80	(0.22, 2.89)
Child (aged≤15)	54	1.00		1.00		1.00		1.00	
Adult (aged 16+)	291	1.75	(0.43, 7.16)	0.59	(0.31, 1.15)	1.40	(0.56, 3.53)	1.28	(0.36, 4.60)
Female	211	1.00		1.00		1.00		1.00	
Male	134	1.10	(0.52, 2.33)	0.87	(0.51, 1.47)	0.76	(0.39, 1.48)	0.99	(0.38, 2.58)
Not vaccinated	308	1.00		1.00		1.00		1.00	
Vaccinated in past 1 year	37	0.46	(0.07, 2.98)	1.42	(0.72, 2.79)	1.30	(0.55, 3.08)	0.63	(0.10, 4.07)
Child (aged≤15) index	52	1.00		1.00		1.00		1.00	
Adult (aged 16+) index	70	0.51	(0.18, 1.43)	0.83	(0.42, 1.66)	0.82	(0.36, 1.87)	0.55	(0.16, 1.84)
Female index	68	1.00		1.00		1.00		1.00	
Male index	54	0.80	(0.30, 2.13)	0.95	(0.48, 1.88)	0.79	(0.35, 1.80)	1.44	(0.43, 4.85)

* Clinical influenza definition 1 is fever≥38°C or at least 2 of headache, runny nose, sore throat, aches or pains in muscles or joints, cough, or fatigue. Clinical influenza definition 2 is at least 2 of fever≥37.8°C, cough, headache, sore throat, aches or pains in muscles or joints. Clinical influenza definition 3 is the standard CDC classification of fever≥37.8°C plus cough or sore throat.

† OR = odds ratio.

### Ancillary analyses

A total of 24 index subjects were prescribed antivirals: 12 oseltamivir and 12 amantadine. By excluding these 24 households, the overall laboratory and clinical secondary attack ratios increased to 6.4% and 20%, 12% and 5% respectively, while the adjusted odds ratios of the intervention effects were similar (data not shown). Only three laboratory-confirmed secondary cases (4.5%) were observed in the 67 household contacts of the 24 index cases prescribed antivirals.

The 21 laboratory-confirmed secondary cases recorded a variety of clinical symptoms and 4 (19%) secondary cases were asymptomatic; all 4 asymptomatic cases were confirmed by viral culture. Of the three case definitions of clinical influenza, the second definition (based on [19]) had slightly higher discriminatory ability, with area under ROC curve 0.74, compared to the gold standard of laboratory outcome, whereas our original per protocol definition and the CDC definition had lower areas under the curve since the former was less specific while the latter was more specific but much less sensitive compared to laboratory-confirmed influenza (Appendix Table S1).

In terms of adherence, 45% (21%) of index subjects (household contacts) in the face mask arm reported wearing a mask often or always during the follow-up period, compared to 30% (1%) and 28% (4%) in the control and hand hygiene arms, respectively. The higher reported compliance in index subjects in the face mask group compared to household contacts was validated when at the final home visits the index subjects had used a median of 12 masks (inter-quartile range, IQR: 6, 18) whereas household contacts had only used a median of 6 (IQR: 1, 20); these include the mask worn and then disposed of by each individual as part of the demonstration and teaching during the initial home visit. A total of 63% (41%) of index subjects (household contacts) in the hand hygiene arm reported washing their hands often or always after sneezing, coughing or cleaning their nose compared to 31% (27%) and 63% (47%) in the control and face mask arms. In the hand hygiene group, households used a median of 56 g (IQR: 27 g, 93 g) of alcohol from the automatic sanitizer, and a median of 88 g (IQR: 63 g, 149 g) of liquid hand soap, while regarding the individual bottles of alcohol hand rub index subjects used a median of 7 g (IQR: 2 g, 13 g) and household contacts used a median of 5 g (IQR: 1 g, 12 g).

### Adverse events

There were no reported adverse events, including allergic reactions to the intervention measures or other conditions requiring medical attention.

## Discussion

If an influenza pandemic emerges, the likely limited supply of antivirals and vaccines will mean that non-pharmaceutical interventions have a major role to play in mitigating disease spread [11], [12]. While conventional wisdom proposes that hand hygiene [8], and perhaps surgical masks [26], could be effective measures to reduce household transmission of influenza, all available data have so far been derived from at best observational settings and mostly based on anecdotal evidence rather than controlled trials [7], [8], [27]. Our study is the first reported community-based randomized trial of these interventions specifically against influenza, with laboratory-confirmed outcomes.

Strengths of our study design include the randomized allocation of interventions, the laboratory-based outcome measures, and our demonstrated ability to observe secondary infections with the implied potential to detect reduction in secondary attack ratios. Whereas the present study was not powered to assess the relative efficacy of the interventions, it has proved successful in demonstrating the feasibility of our study design and the local characteristics of influenza transmission. The present findings have facilitated the planning of a subsequent larger study, described in more detail in Protocol S1.

Although we found little effect of the interventions in preventing household transmission, our study was underpowered. Nevertheless, our point estimates are close to null, strongly suggesting true equipoise until a definitive randomized trial with sufficient power (i.e. a much larger sample size) rigorously tests the relative efficacy of these interventions. A larger study will also allow us to explore in more detail the transmission dynamics of influenza in households including finer age stratifications and transmission within and between different age groups, which was not possible in the current study.

We observed generally low adherence to interventions. More than one in four household contacts in the face mask group did not wear a surgical mask at all during the follow-up period. Adherence to the face mask intervention was higher in the index subjects, likely due to their intention to reduce the probability of infecting other household members and possibly because of the recent memory of SARS in 2003, during which the majority (76%) of the general public reported that they wore face masks in public, and most engaged in numerous protective practices [28], [29]. However more than one in four index cases in the control and hand hygiene intervention arms reported wearing masks at home of their own accord, thereby contaminating this intervention.

While self-reported hand-hygiene practices were similar across the three groups, we note that contamination of this intervention may be lower firstly because the control and face mask group did not receive the education component on proper hand hygiene, secondly because those groups did not receive the alcohol sanitizer and hand rub. Overall, adherence to the hand hygiene intervention in terms of soap and alcohol use appeared low when benchmarked against rates recommended in health care settings. However we note that a previous randomized community study found that 38% of households used more than 57 g of alcohol hand sanitizer during a 2-week period [30], whereas more than 50% of the households in our study used more than 56 g in 10 days.

Overall, the SAR was lower than we had expected. Only 6% of household contacts developed laboratory-confirmed influenza, whereas 5%–18% of contacts developed clinical influenza, depending on case definitions. This is in contrast to previous studies in France [21], Seattle [31] and other places [19], where SARs were approximately 25% (laboratory-confirmed influenza in the latter two studies). There could be a number of reasons for this. First, there has not been significant antigenic drift in the predominant circulating strains of influenza viruses in recent years, potentially resulting in higher levels of pre-existing immunity among our study population. Secondly, our inclusion criteria specified that an index subject should be the only member of their household to be suffering from ILI, and no other household contacts should have experienced ILI in the past 14 days, to ensure that the index is a true index within the household. However, the latter condition may have biased our recruitment towards households where some members were already immune from infection, since among households where all contacts were susceptible there might be a greater possibility of secondary cases being observed prior to the index case presenting to their primary care provider 1–2 days after symptom onset (Figure 2a). However the French study used similar inclusion criteria and found a much higher SAR [21]. Antiviral prescriptions for index subjects followed with home visits appears to affect transmission as would be expected [19], where there was a relative reduction in the SAR of approximately 30% albeit based on a limited sample size. Vaccination of household contacts might also have reduced the risk of secondary infection (Table 3). Finally, environmental or behavioral differences could lead to differing secondary transmission rates in our study, for example differences in use of air conditioning, high background use of face masks, or differences in the amount of time spent with family members at home. We did not collect the relevant data in the present study however; future studies should consider these externalities. Results from other settings with a similar design would be helpful in assessing, at least qualitatively, these respective effects.

The variability in clinical SARs depending on the choice of case definition has been noted in previous studies [21], [32], [33]. Influenza infection is associated with a wide spectrum of symptoms and severities, and in our study 4 (19%) of the 21 laboratory-confirmed secondary cases were asymptomatic. On the other hand, only 10 (48%) of laboratory-confirmed secondary cases reported fever ≥38°C. With such a range of symptoms caused by influenza, and when infections with other circulating upper respiratory viruses cause similar symptoms, collectively referred to as influenza-like-illnesses, it is difficult to find a single case definition which is highly sensitive and also highly specific for influenza virus. With a small sample size it is not possible here to derive clinical prediction rules [33]–[38], however we compared three alternative case definitions and found that the most predictive (with highest area under the ROC curve) was at least two of the following signs and symptoms: fever ≥37.8°C, cough, headache, sore throat, aches or pains in muscles or joints (Appendix Table S1). While the clinical SARs rely on self-reported symptoms, the diaries were checked for completeness and accuracy by trained nurses during home visits every 3 days. The proportion of asymptomatic infection in our study was lower than might have been expected (i.e. closer to 50%) based on earlier studies with paired serology [31], perhaps suggesting that we might have missed some infections when assessing the secondary outcomes of clinical influenza. The corollary is that the true secondary infection rate might well have been higher than estimated (and estimable) by our SARs.

The dropout was higher than anticipated; all subjects were advised of the study requirements and gave informed consent before being recruited into the study (and tested by rapid influenza test without charge), but 35% of randomized subjects/households refused to allow any home visits. These decisions were independent of the allocated intervention, since the interventions were only revealed during the first home visit. Dropout was higher among the group randomized with a negative result on the rapid diagnostic test (after June 1, 2008), perhaps because subjects interpreted their negative result as indicating they did not have influenza thus did not require follow-up. A negative rapid test result does not rule out influenza virus infection [39], and we chose to randomize such subjects to allow wider generalizability in terms of including index subjects with a likely greater range of influenza viral shedding profiles albeit with the limitation that some index subjects might have been infected with a different pathogen; in the latter case those households would be unnecessarily followed up since only households with index subjects with confirmed influenza (by viral culture or RT-PCR) were included in the final analyses. We found that dropout rates were lower when the index subject wasaged 15 years or younger (Table 1) perhaps because the accompanying parent would have also given immediate consent.

Other limitations of our study design include the potential bias from recruiting symptomatic subjects, resulting in three distinct effects. First, the use of a point-of-care test to detect influenza virus infection, ensuring that the majority of followed-up households will include an index case with laboratory-confirmed influenza (98% in our study), could also preferentially detect those potential recruits with higher viral shedding and subjects with lower levels of viral shedding would be more likely to receive a false negative rapid test results, and not be recruited. However we note that statistical power would be generally increased if index cases were more infectious since we might therefore observe more secondary transmission; the limitation here relates more to generalizability. Secondly, our design results in an unavoidable delay between onset of symptoms in the index subject and the application of the intervention (Figure 2c). If a significant amount of influenza transmission occurred prior to the intervention, we might have underestimated the efficacy of the non-pharmaceutical interventions or lacked the statistical power to find significant differences. In our analyses we investigated the SARs for those households where the intervention was applied within 36 hours of symptom onset but there was no indication of greater efficacy in this subgroup. Thirdly, there is the potential for recruited households to be biased towards including household contacts with pre-existing immunity, as discussed above. An alternative approach would have been to randomize a much larger cohort of initially uninfected households, who were then followed throughout an influenza season. However such a longitudinal study would require greater resources by several orders of magnitude than the one proposed here, due to the low attack rate of influenza.

In conclusion, there remains a serious deficit in the evidence base of the efficacy of non-pharmaceutical interventions. The US Centers for Disease Control and Prevention have awarded grants to study non pharmaceutical interventions in community settings [40], including this study. Other funded study designs include symptom-based recruitment (as in our study) and longitudinal studies of initially uninfected cohorts, in children and adults and in various settings including households, schools and student halls of residences. We eagerly anticipate that conclusive evidence will become available as these studies proceed in the coming months, finally allowing empirically-driven pandemic planning.

## Supporting Information

Table S1 Performance of alternative definitions of clinical influenza versus the gold standard of laboratory-confirmed influenza infection in household contacts.
(0.03 MB DOC)

Figure S1 Study recruitment versus local influenza activity. a) daily recruitment rate (patients per working day); b) Local sentinel surveillance data of influenza-like-illness (number of ILI consultations per 1000 consultations) by the Centre for Health Protection; c) rate of positive influenza isolations among specimens submitted to the WHO reference laboratory of Queen Mary Hospital, Hong Kong.
(1.23 MB EPS)

Text S1 Appendix with additional details of laboratory procedures
(0.02 MB PDF)

Protocol S1 Trial protocol
(0.17 MB PDF)

Checklist S1 CONSORT checklist
(0.03 MB PDF)

## References

[1] Proença-Módena JL, Macedo IS, Arruda E (2007). H5N1 avian influenza virus: an overview.. Braz J Infect Dis.

[2] Beigel JH, Farrar J, Han AM, Hayden FG, Hyer R (2005). Avian influenza A (H5N1) infection in humans.. N Engl J Med.

[3] Ungchusak K, Auewarakul P, Dowell SF, Kitphati R, Auwanit W (2005). Probable person-to-person transmission of avian influenza A (H5N1).. N Engl J Med.

[4] Kandun IN, Wibisono H, Sedyaningsih ER, Yusharmen, Hadisoedarsuno W (2006). Three Indonesian clusters of H5N1 virus infection in 2005.. N Engl J Med.

[5] Stohr K, Esveld M (2004). Public health. Will vaccines be available for the next influenza pandemic?. Science.

[6] Lipsitch M, Cohen T, Murray M, Levin BR (2007). Antiviral Resistance and the Control of Pandemic Influenza.. PLoS Medicine.

[7] Aledort JE, Lurie N, Wasserman J, Bozzette SA (2007). Non-pharmaceutical public health interventions for pandemic influenza: an evaluation of the evidence base.. BMC Public Health.

[8] Aiello AE, Coulborn R, Perez V, Larson EL (2008). Effect of Hand Hygiene on Infectious Disease Risk in the Community Setting: A Meta-Analysis.. Am J Public Health (in press).

[9] Jefferson T, Foxlee R, Mar CD, Dooley L, Ferroni E (2008). Physical interventions to interrupt or reduce the spread of respiratory viruses: systematic review.. BMJ.

[10] Epstein JM, Goedecke DM, Yu F, Morris RJ, Wagener DK (2007). Controlling Pandemic Flu: The Value of International Air Travel Restrictions.. PLoS ONE.

[11] World Health Organization (WHO) Writing Group (2006). Nonpharmaceutical Interventions for Pandemic Influenza, International Measures.. Emerg Infect Dis.

[12] World Health Organization (WHO) Writing Group (2006). Nonpharmaceutical Interventions for Pandemic Influenza, National and Community Measures.. Emerg Infect Dis.

[13] Wong CM, Chan KP, Hedley AJ, Peiris JS (2004). Influenza-associated mortality in Hong Kong.. Clin Infect Dis.

[14] Monto AS (1999). Individual and community impact of influenza.. Pharmacoeconomics 16 Suppl.

[15] Molinari NA, Ortega-Sanchez IR, Messonnier ML, Thompson WW, Wortley PM (2007). The annual impact of seasonal influenza in the US: measuring disease burden and costs.. Vaccine.

[16] Campbell MK, Elbourne DR, Altman DG, group C (2004). CONSORT statement: extension to cluster randomised trials.. BMJ.

[17] World Medical Association (2000). World Medical Association Declaration of Helsinki: ethical principles for medical research involving human subjects.. JAMA.

[18] Larson EL (1995). APIC guideline for handwashing and hand antisepsis in health care settings.. Am J Infect Control.

[19] Monto AS, Pichichero ME, Blanckenberg SJ, Ruuskanen O, Cooper C (2002). Zanamivir prophylaxis: an effective strategy for the prevention of influenza types A and B within households.. J Infect Dis.

[20] Babcock HM, Merz LR, Fraser VJ (2006). Is influenza an influenza-like illness? Clinical presentation of influenza in hospitalized patients.. Infect Control Hosp Epidemiol.

[21] Viboud C, Boelle PY, Cauchemez S, Lavenu A, Valleron AJ (2004). Risk factors of influenza transmission in households.. Br J Gen Pract.

[22] Donner A, Klar N (2000). Design and analysis of cluster randomization trials in health research.

[23] Liang K-Y, Zeger SL (1986). Longitudinal data analysis using generalized linear models.. Biometrika.

[24] Pepe MS (2003). The Statistical Evaluation of Medical Tests for Classification and Prediction.

[25] R Development Core Team (2004). R: A language and environment for statistical computing.

[26] Tellier R (2006). Review of aerosol transmission of influenza A virus.. Emerg Infect Dis.

[27] Jefferson T, Foxlee R, Mar CD, Dooley L, Ferroni E (2007). Physical interventions to interrupt or reduce the spread of respiratory viruses: systematic review.. BMJ (in press).

[28] Lo JY (2005). Respiratory Infections during SARS Outbreak, Hong Kong, 2003.. Emerg Infect Dis.

[29] Leung GM, Ho LM, Chan SK, Ho SY, Bacon-Shone J (2005). Longitudinal assessment of community psychobehavioral responses during and after the 2003 outbreak of severe acute respiratory syndrome in Hong Kong.. Clin Infect Dis.

[30] Sandora TJ, Taveras EM, Shih MC, Resnick EA, Lee GM (2005). A randomized, controlled trial of a multifaceted intervention including alcohol-based hand sanitizer and hand-hygiene education to reduce illness transmission in the home.. Pediatrics.

[31] Fox JP (1980). Viruses in families.

[32] King JC, Stoddard JJ, Gaglani MJ, Moore KA, Magder L (2006). Effectiveness of school-based influenza vaccination.. N Engl J Med.

[33] Monto AS, Gravenstein S, Elliott M, Colopy M, Schweinle J (2000). Clinical signs and symptoms predicting influenza infection.. Arch Intern Med.

[34] Zambon M, Hays J, Webster A, Newman R, Keene O (2001). Diagnosis of influenza in the community: relationship of clinical diagnosis to confirmed virological, serologic, or molecular detection of influenza.. Arch Intern Med.

[35] Friedman MJ, Attia MW (2004). Clinical predictors of influenza in children.. Arch Pediatr Adolesc Med.

[36] Ohmit SE, Monto AS (2006). Symptomatic predictors of influenza virus positivity in children during the influenza season.. Clin Infect Dis.

[37] Carrat F, Tachet A, Rouzioux C, Housset B, Valleron AJ (1999). Evaluation of clinical case definitions of influenza: detailed investigation of patients during the 1995–1996 epidemic in France.. Clin Infect Dis.

[38] Call SA, Vollenweider MA, Hornung CA, Simel DL, McKinney WP (2005). Does this patient have influenza?. JAMA.

[39] Uyeki TM (2003). Influenza diagnosis and treatment in children: a review of studies on clinically useful tests and antiviral treatment for influenza.. Pediatr Infect Dis J.

[40] Morse SS, Garwin RL, Olsiewski PJ (2006). Public health. Next flu pandemic: what to do until the vaccine arrives?. Science.

