# Supplementary material for: Preliminary Findings of a Randomized Trial of Non-Pharmaceutical Interventions to Prevent Influenza Transmission in Households
Source: PLoS One. 2008 May 7;3(5):e2101. doi: 10.1371/journal.pone.0002101 (PMC2364646; doi:10.1371/journal.pone.0002101)
Supplement: Table S1 — Performance of alternative definitions of clinical influenza versus the gold standard of laboratory-confirmed influenza infection in household contacts. (0.03 MB DOC) [file pone.0002101.s001.doc]

## Table S1: Performance of alternative definitions of clinical influenza versus the gold standard of laboratory-confirmed influenza infection in household contacts.

| **Definition** | **Sensitivity** | **Specificity** | **Area under ROC (95% CI*)** |
| --- | --- | --- | --- |
| Clinical influenza definition 1^†^ | 0.57 | 0.81 | 0.69 (0.59, 0.80) |
| Clinical influenza definition 2^†^ | 0.57 | 0.91 | 0.74 (0.62, 0.84) |
| Clinical influenza definition 3^†^ | 0.48 | 0.97 | 0.73 (0.61, 0.83) |

*95% confidence interval for areas under ROC estimated by bootstrapping with 1000 resamples.

^†^ Clinical influenza definition 1 is fever≥38°C or at least 2 of headache, runny nose, sore throat, aches or pains in muscles or joints, cough, or fatigue. Clinical influenza definition 2 is at least 2 of fever≥37.8°C, cough, headache, sore throat, aches or pains in muscles or joints. Clinical influenza definition 3 is the standard CDC classification of fever≥37.8°C plus cough or sore throat.
