# Supplementary material for: Preliminary Findings of a Randomized Trial of Non-Pharmaceutical Interventions to Prevent Influenza Transmission in Households
Source: PLoS One. 2008 May 7;3(5):e2101. doi: 10.1371/journal.pone.0002101 (PMC2364646; doi:10.1371/journal.pone.0002101)
Supplement: Text S1 — Appendix with additional details of laboratory procedures (0.02 MB PDF) [file pone.0002101.s003.pdf]

Laboratory Methods

Viral culture

Madin-Darby canine kidney (MDCK) cell monolayers in culture tubes were inoculated with 200 µl of the nasal swabs-virus transport medium suspension and the cells were maintained in serum-free minimum essential medium (MEM, Gibco, N.Y., USA) containing tosylsulfonyl phenylalanyl chloromethyl ketone-treated trypsin (2µg/ml) (Sigma, St. Louis, MO), and incubated at 33°C for 7 days (MDCK cells). They were examined daily for cytopathic effect, and immunofluoresence was done on fixed cell smears when CPE appeared or at the end of the incubation period [1].

RT-PCR

Total nucleic acid was extracted from the specimens using NucliSens easyMAG extraction system (bioMerieux, Netherlands) according to manufacturer's instructions. Twelve µl of extracted nucleic acid was used to prepare cDNA by Invitrogen Superscript III kit with random primer as described previously [2] For influenza A or B virus, 2µl of cDNA was amplified in LightCycler with a total volume of 20µl reaction containing FastStart DNA Master SYBR Green I Mix reagent kit (Roche Diagnostics GmbH, Germany), 4.0mM $MgCl_2$ and 0.5µM of each primer. The forward primer (5'-CTTCTAACCGAGGTCGAAACG-3') and the reverse primer (5'-GGCATTTTGGACAAAKCGTCTA-3) were used for amplification corresponding to the M gene of influenza A [1]. Cycling conditions were as follows: an initial denaturation at 95ºC for 10 minutes, followed by 40 cycles of 95ºC for 10 seconds, 60ºC for 3 seconds, 72ºC for 12 seconds with ramp rates of 20ºC/second. For influenza B, forward (5'- GGGATATACGTAATGTGTTGT) and reverse (5'-GCACTGCCTGCTGTACACTT) primers was used to amplify a 489bp product corresponding to the nonstructural protein [3]. Cycling conditions were as follows: an initial denaturation at 95ºC for 10 minutes, followed by 45 cycles of 95ºC for 10 seconds, 55ºC for 5 seconds, 72ºC for 20 seconds with ramp rates of 20ºC/second. A series of dilutions were prepared to generate calibration curves and run in parallel with the test samples. At the end of the assay, PCR products were subjected to a melting curve analysis to determine the specificity of the assay.

References

1. Chan KH, Peiris JSM, Lim W, Nicholls JM, Chiu SS. (2008) Comparison of nasopharyngeal flocked swabs and aspirates from pediatric patients for rapid diagnosis of respiratory viruses. J Clin Virol (in press).

2. Peiris JSM, Tang WH, Chan KH, Khong PL, Guan Y, et al. (2003) Children with respiratory disease associated with metapneumovirus in Hong Kong. Emerg Infect Dis 9: 628-633.

3. Gruteke P, Glas AS, Dierdorp M, Vreede WB, Pilon JW, et al. (2004) Practical implementation of a multiplex PCR for acute respiratory tract infections in children. J Clin Microbiol 42: 5596-5603.
