# Supplementary material for: Preliminary Findings of a Randomized Trial of Non-Pharmaceutical Interventions to Prevent Influenza Transmission in Households
Source: PLoS One. 2008 May 7;3(5):e2101. doi: 10.1371/journal.pone.0002101 (PMC2364646; doi:10.1371/journal.pone.0002101)
Supplement: Protocol S1 — Trial protocol (0.17 MB PDF) [file pone.0002101.s004.pdf]

A Randomised Controlled Trial of Face Masks and Hand Hygiene in Reducing Influenza Transmission in Households

Protocol number GML001.5 (10 December 2007)

Investigators:

School of Public Health, The University of Hong Kong, Hong Kong
Professor G.M. Leung (**PI**)
Dr B.J. Cowling
Dr L.M. Ho

Department of Microbiology, The University of Hong Kong, Hong Kong
Professor J.S.M. Peiris
Dr K.H. Chan

Centre for Health Protection, Hong Kong
Dr R. Yung

Department of Microbiology, Queen Mary Hospital, Hong Kong
Dr W.H. Seto

Division of Global Migration and Quarantine, National Center for Preparedness, Detection and Control of Infectious Diseases, CDC, Washington, Seattle, USA
Dr P.M. Houck

Influenza Division, CDC, Atlanta, Georgia, USA
Dr T.M. Uyeki

St Paul's Hospital, Hong Kong
Dr. P. Lee

Hong Kong Sanatorium
Dr. B. Chiu

Pamela Youde Nethersole Eastern Hospital, Hong Kong
Dr Y.F. Choi

Table of contents:

1. Title 3
2. Background Information 3
3. Trial Objectives and Purpose 5
4. Trial Design 5
5. Selection and Withdrawal of Subjects 10
6. Treatment of Subjects 11
7. Assessment of Efficacy 12
8. Assessment of Safety 13
9. Statistical Analysis 13
10. Direct Access to Source Data 18
11. Ethics 18
12. Data Handling and Record Keeping 18
13. Financing and Insurance 18
14. Publication Policy 18
15. References 19

1. Title

A randomised controlled trial of face masks and hand hygiene in reducing influenza transmission in households.

2. Background Information

Relevant definitions:
Index case: the first subject to be infected with influenza in a household.
Household contact: any person living in the same household as the index case.
Secondary attack ratio (SAR): the proportion of household contacts of an index case who subsequently become infected with influenza.
Hand washing: a process for the removal of soil and transient microorganisms from the hands [1].
Hand antisepsis: a process for the removal or destruction of transient microorganisms [1].

a) Name and description of the investigational products and interventions

The investigational products include surgical face masks, liquid hand soap and hypoallergenic waterless alcohol-based hand cleanser with emollient. The interventions will incorporate distribution of these investigational products and education on their proper use.

b) Relevant prior studies

There is currently concern about the possibility of an impending emerging influenza pandemic. In the event of such, a limited number of interventions would be available to reduce and control the spread of the disease and thus the resulting morbidity and mortality [2, 3]. Antiviral drugs could be used subject to availability, although their effectiveness against the novel pandemic strain is uncertain and resistance could develop quickly with large-scale use. Furthermore a vaccine specific to the pandemic strain would take optimistically, given current technology and production capacity, at least 6 months to develop and mass produce [4]. In addition to vaccination and targeted antiviral prophylaxis, other population-level social distancing measures such as school and workplace closures and travel restrictions are likely to be somewhat effective in reducing influenza transmission in the community [5, 6], but implementation on a prolonged basis and with repeated waves of the pandemic could be difficult. Household-based quarantine and isolation will likely be effective in mitigating the impact of a pandemic [5-7]. There is however considerable uncertainty about the efficacy of some non-pharmaceutical interventions at the personal level including face masks and hand hygiene. Our proposed study, to assess the efficacy of masks and hand-hygiene for influenza control, is a direct response to the World Health Organization’s recent call for urgent research on the efficacy of non-pharmaceutical public health interventions [3].

In addition to pandemic preparedness, knowledge about the efficacy of masks and hand hygiene would also be important for inter-pandemic influenza control. In western temperate and regional subtropical countries, influenza is a major source of

morbidity and mortality during the seasonal periods of epidemic circulation [8, 9], and a number of measures are typically taken to try to reduce transmission in hospitals and elderly care homes [1]. However there are few data on the efficacy of such measures in the household, although household transmission is thought to be one of the most important settings for the community transmission of influenza [10].

Previous studies have tentatively suggested that prophylaxis with amantadine, rimantadine, and the neuraminidase inhibitors oseltamivir (Tamiflu®) and zanamivir (Relenza®) are effective in reducing influenza transmission in households [11, 12]. Vaccination is known to be an effective preventative measure [13] provided that the at least one of the vaccine strains closely matches the circulating strain [14].

Protective interventions at the personal level to reduce influenza transmission such as wearing masks and improving hand hygiene are often recommended [15] but few studies have investigated the efficacy of these measures outside nosocomial settings in a rigorous manner. Influenza is thought to be mainly transmitted through airborne droplet nuclei [16] but to a significant extent also spread by hand and surface transfer [17]. Some studies have suggested that transmission of upper respiratory tract infections was reduced after household-based hygiene interventions [18-20], while a recent case-control study has suggested that masks and hand-washing may have been effective in reducing the transmission of SARS in a hospital setting [21]. A recent population study has suggested that improved hygiene measures and decreased community mixing during the SARS outbreak in Hong Kong resulted in reduced incidence of respiratory viral infections [22].

The results of this study will have important implications for influenza prevention both in a pandemic and in interpandemic periods. Quantitative estimates of the efficacy of non-pharmaceutical interventions will inform resource allocation under pandemic preparedness plans.

c) Summary of known and potential risks and benefits

Wearing masks may diminish the rate of influenza transmission by reducing the amount of virus-containing droplet nuclei entering the surrounding area after leaving the mouth and nose of an infected subject. When worn by a non-infected subject in the presence of infected airborne droplets, surgical masks may reduce the amount of infected droplets inhaled and thereby reduce the chances of infection. However the degree to which a surgical mask can reduce airborne transmission is difficult to quantify given the lack of prior research in this area, and the expected benefit is uncertain. There are few apparent risks of wearing a mask, perhaps the greatest risk being that the mask engenders a feeling of overconfidence in the ability of the mask to prevent infection, leading to riskier activity (e.g. sitting closer to family members at mealtimes) than might have taken place if the mask were not worn, and thus an increased rather than decreased risk of influenza transmission.

Proper hand hygiene is thought to reduce community transmission of some viral infections including rhinoviruses and RSV [10], but the specific effect of hand hygiene on influenza has not been quantified. Again there are few apparent risks of proper hand hygiene, perhaps the greatest being the detrimental effects on skin of frequent hand washing, particularly with alcohol-based products although most of

these contain emollients to buffer against excessive drying. To try to mitigate these effects as much as possible we will only supply an alcohol-based hand cleanser which includes emollients, and encourage study participants to be wary of skin irritation. The other very rare potential adverse consequence, as with all topical applications, is allergic reaction. We will use a hypoallergenic product and exclude participants who have a known allergy to alcohol or additive components of the alcohol handrub deployed.

d) Description of and justification for the route of administration and treatment period

The face masks and hand washing interventions will include an intensive counselling session to describe and demonstrate proper use of the respective hygiene aids.

Given that the index case could be symptomatic for a further 5 days, and asymptomatic incubation of the disease in household contact could take 1-2 days before symptoms appear, we propose that the hygiene measures should be maintained for at least 7 days.

e) Statement of proper conduct

The trial will be conducted in compliance with this protocol, GCP, and the applicable regulatory requirements.

f) Description of the population to be studied

The population studied are households in Hong Kong containing three or more individuals where at least one individual is suspected to be infected with influenza (ascertained either by meeting specific symptom criteria or by a positive result on a rapid diagnostic test, see 4(d)) and where no other household members have experienced symptoms of influenza-like-illness in the preceding two weeks.

3. Trial Objectives and Purpose

To quantify the efficacy of face masks and/or hand hygiene in reducing household transmission of influenza.

4. Trial Design

a) Primary endpoint

The primary outcome measure is the SAR i.e. the proportion of household contacts with laboratory-confirmed influenza during the study period (follow-up period of 10/7 days in pilot/main study). Inference will be made at the individual rather than household level, adjusting for the potential within-household correlation.

b) Trial design

The main study will follow a parallel design with three intervention arms (i.e. routine health education only, hand hygiene only, masks and hand hygiene). Households will be randomly assigned to one of the three interventions although intention-to-treat analysis will be at the individual level; therefore this is a cluster-randomised trial design. The pilot study will have three arms (mask only, hand hygiene only, routine health education only).

Subject recruitment will take place at selected government general outpatient clinics, group/managed practices, public hospital emergency rooms, private hospital outpatient departments, and private primary care clinics throughout Hong Kong, Kowloon and the New Territories.

For the pilot study we propose to recruit 500 individuals with ILI symptoms and apply the QuickVue rapid diagnostic test, so that we can follow-up a maximum of 200 index cases with a positive test result, and their households. We will stop recruiting as soon as we have 200 influenza-positive index cases, and we will stop recruiting after we have used 1,000 rapid diagnostic tests even if we have fewer than 200 influenza-positive index cases. Each household will be randomized to receive one of the three interventions, and all household contacts will be followed up. Details of the power calculation to justify this sample size are given below. Given an average household size of 3.8, a study of 200 households will involve the enrolment of a total of 760 individuals (200 index cases and 560 household contacts).

For the main study we propose to recruit approximately 6,000 individuals with symptoms of influenza-like illness (ILI) and follow-up an anticipated 1,200 index cases who meet specific criteria (in most cases a positive rapid diagnostic test result, or in some cases those meeting symptom based criteria – further described in 4(d)) and their households. Each household will be randomized to receive one of three interventions, and all household contacts will be followed up. Given an average household size of 3.8, a study of 900 households (from 1,200 randomized households after an anticipated 25% dropout) will involve the enrolment of a total of 3,420 individuals (900 index cases and 2,520 household contacts). Details of the power calculation to justify this sample size are given in 9(c) below.

c) Measures to minimise bias

A cluster-randomisation design has been chosen because simple individual randomisation of household contacts would almost certainly result in cross-contamination between family members who are assigned to different intervention arms. This cluster randomisation design requires a larger sample size to allow for potential within-household variability in SAR.

Given the nature of the interventions, participants will be un-blinded to the intervention received although they will be blinded to the nature of the other interventions. The same will apply to our research nurses who will be educating participating households on uses of assigned interventions and prevention of influenza transmission. Nurses will initially be randomly assigned to administering one of the three interventions and will receive training relevant to their assigned intervention only, hence avoiding potential cross-contamination by nurses.

When the details of a new index case are uploaded to the online database by fax to the trial manager, a unique identifier will be assigned. A pre-specified table of random numbers will be used to assign one of the three interventions to the household of the index case. Therefore the randomised intervention will be unknown to the doctor at or after the time of recruitment to minimise allocation and ascertainment biases respectively.

d) Criteria for further follow-up

In the majority of recruiting sites, the criteria for further household follow-up will be a positive result for influenza A or B using the QuickVue rapid diagnostic test on a nose and throat swab (Quidel Corp, San Diego, CA) which has a reported sensitivity of 79% and specificity of 92% for influenza A or B [23]. If the QuickVue test is positive and informed consent is obtained, the index case and their household will be further followed up with home visits as described above. We will calculate the sensitivity and specificity of the rapid diagnostic test in our study setting, using viral culture or PCR of a nose and throat swab as the gold standard.

In a small number of recruiting sites at defined periods during the pilot and main studies we will use a symptom-based criteria to determine eligibility for further follow-up; specifically we will enrol subjects and their households for further follow-up if they present with at least two of the following symptoms: fever≥37.8°C (≥38°C in the pilot study); cough; headache; sore throat; pain in muscles or joints [24].

In a small number of recruiting sites at defined periods during the main study we will use an alternative rapid diagnostic test, namely the HX Diagnostics Influenza A+B test (HX Diagnostics, San Francisco, CA) which is based on 3rd generation lateral flow immunoassay technology and may be more sensitive and specific than the QuickVue test. In this case, subjects with a positive result on the HX Diagnostics Influenza A+B test would be eligible for further follow up as described above. We will calculate the sensitivity and specificity of the rapid diagnostic test in our study setting, using viral culture or PCR of a nose and throat swab as the gold standard.

See also 7(c).

e) Trial interventions

See 6(a).

f) Home visits

Following randomization, an immediate home visit will be scheduled (to take place within at most 36 hours, and ideally within 12 hours) to implement the intervention. During the home visit by a trained nurse, the purpose of the study will be explained to all household contacts and their consent obtained. Consent for children aged 17 years or younger will be obtained from their parents. Assent will also be obtained for children aged between 7 through 17. The nurse will then collect details on household composition, risk perceptions, attitudes and beliefs on influenza (questionnaire Q2, appendix), and take a nose swab and a throat swab from each household contact, except for asymptomatic children under the age of 2. Due to concerns about

difficulties of taking nose and throat swabs from infants, for household members who are under 2 years of age only information on clinical symptoms will therefore be collected unless they are symptomatic. For participants who are symptomatic, a nose and a throat swab will be collected regardless of their age. The swabs will later be tested to confirm the absence of influenza in any household contact at baseline. The nurse will provide and describe proper use of a free tympanic thermometer, and the daily symptom diaries. Finally, the nurse will administer the standardized intervention through intensive counselling, and demonstration of proper wearing of masks or hand washing.

Three and six days (±1 day) after the initial home visit, a trained nurse will revisit each household. During the visit, the nurse will take nose swabs and throat swabs from the index case and all household contacts, except for children under 2 years of age who are asymptomatic, and collect the symptom diaries (questionnaire Q3, appendix) from each household contact. During the final visit (day 6), the nurse will ask the household members to complete a final questionnaire (Q4, appendix) and assess their adherence to the interventions (questionnaire Q5, appendix).

g) Trial period

The 8-week pilot study will take place from January to April 2007. The exact starting and stopping dates of patient recruitment will not be fixed in advance. Recruitment will begin after the start of the annual influenza peak season (typically Feb/Mar) has been confirmed by the Department of Microbiology, HKU, and will continue until the prespecified sample size is reached. The 39-week main study will take place from January to September 2008.

h) Stopping rules

Individuals may at any time decide to stop participating if they wish, without prejudice or any adverse consequences. There are no formal rules for stopping the trial early.

i) Maintenance of trial treatment randomisation codes and procedures for breaking codes.

When the details of a new index case are uploaded to the online database, a unique identifier will be assigned. A table of random numbers will be generated by the trial statistician prior to the start of the trial, and this will be used to assign one of the three interventions to the household of the index case. Randomisation codes will not be used since for the first home visit it is necessary for the nurse to know which of the interventions has been assigned.

Randomisation codes will be masked from those assessing the outcomes.

k) The identification of any data to be recorded directly on the case record forms (i.e. no prior written or electronic record of data) and to be considered source data.

See questionnaires Q1-Q5 (appendix) for the source data that will be recorded in this study. Note that we will not access subjects' medical records.

l) Prevention of influenza in study nurses

Nurses will be offered influenza vaccination prior to the study.

m) Incentives to participate

We will offer each participating household a HK$200 (US$25) supermarket voucher (HK$150 / US$20 in the pilot study).

n) Centralized collection and storage of specimens

Specimens collected in recruiting clinics will be stored in a 2-8°C refrigerator (overnight, if required). Specimens collected during home visits will be stored at room temperature or if necessary (i.e. in hot weather) in a cool box with at least 2 icepacks immediately after collection. Before the end of the day of a home visit, the study nurse will take any collected samples to the nearest recruitment clinic for storage in a 4°C refrigerator (overnight, if required) or directly to the Department of Microbiology, HKU. Samples stored at 2-8°C in recruiting clinics will be delivered to the Department of Microbiology, HKU as soon as possible, via courier and maintained at 2-8°C en route. Samples will be eluted and cryopreserved at minus 70°C at the destination.

o) Flow chart of main study design

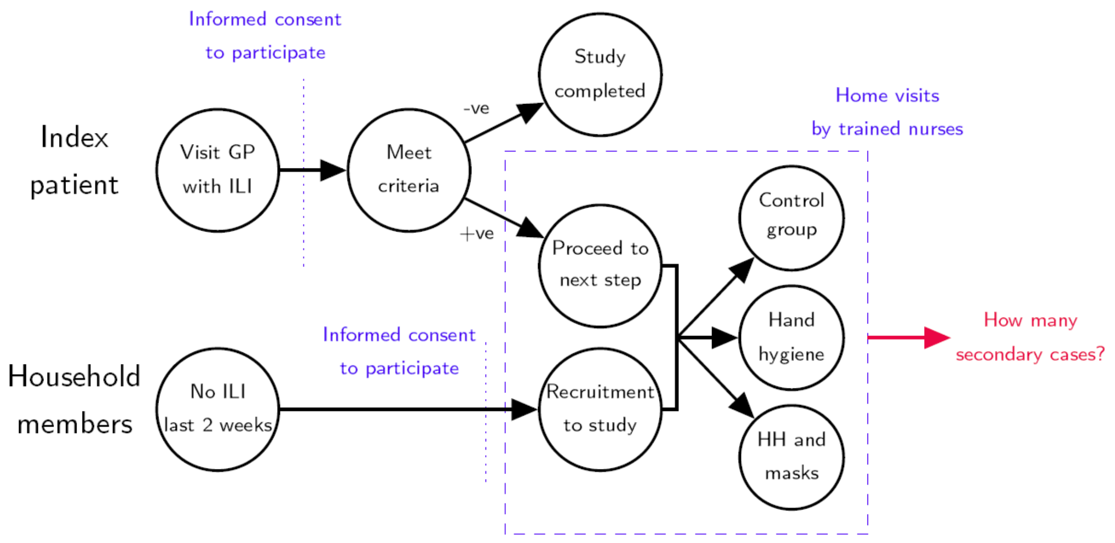

The criteria for further follow-up are discussed in 4(d), in the majority of recruiting sites the criteria will be a positive result on the QuickVue Influenza A+B rapid diagnostic test.

p) Buccal swabs

During the final home visit in the main study we will request buccal swabs from all household members. The samples will be anonymised and then processed by a biotech company specialising in DNA extraction from human samples. These data will be

studied at a later date to investigate possible genetic factors in influenza susceptibility or transmission.

5. Selection and Withdrawal of Subjects

a) Inclusion criteria for index cases and households

Inclusion criteria for index cases are as follows: (1) a Hong Kong resident; (2) reporting ILI symptoms including at least two of fever (recorded fever ≥38°C), cough; nasal congestion; sore throat; headache; runny nose and pains in muscles or joints [24] (3) onset of symptoms within the preceding 48 hours. If these conditions are satisfied, the subject will be approached to determine household eligibility to enrol in the study as below prior to further follow-up as described in 4(d) and 4(f).

Inclusion criteria for households are as follows: The household must contain at least three people including the index case and any domestic helpers, and where no household contacts have had ILI symptoms in the preceding two weeks.

In the main study (January to September 2008), if we are ahead of our recruitment target at any time in March or later, we will consider revising the inclusion criteria to include only subjects with onset of symptoms in the preceding 36 (or 24) hours, since we believe the interventions will be most effective if applied sooner after symptom onset. If implemented, this protocol change would be taken into account in the final analyses.

b) Subject exclusion criteria

There are no exclusion criteria.

c) Subject withdrawal criteria

The entire household will be withdrawn from the study if there is failure to obtain proper informed consent from any one or more individual household contacts for whatever reason. Informed consent will be sought from each individual household contact, or proxy consent from the parents of any individual under the age of 18. Assent will also be obtained for children age between 7 through 17. Households will be withdrawn from the trial if no home visit can be scheduled within 36 hours. Withdrawn households will be replaced to maintain the specified sample size. If one or more household contacts are not present during one of the home visits, we will attempt to reschedule a supplementary home visit to collect clinical specimens, and in the case where the initial home visit was missed we will request signed informed consent and apply interventions as necessary during the supplementary home visit.

In the main study, if a randomized household refuses to allow any home visits, we will request permission to contact them by telephone after 7 days to enquire how long the index case experience symptoms for, and whether any household members reported clinical influenza; if available, these data will allow a simple comparison of households who dropout with those who are successfully followed up.

6. Treatment of Subjects

a) Treatments administered

We will randomize households among three study arms, each of which will include intensive counselling during the first household visit. All recruited households will receive educational pamphlets specific to their assigned intervention arms in English or Chinese or both, as appropriate. The intervention arms are as follows:

> *(i) Control intervention (general health education):*
> This group will receive education about the importance of a healthy diet and lifestyle for boosting the immune system against influenza and other directly transmitted respiratory pathogens leading to ILI symptoms, both in terms of illness prevention and symptom alleviation.
>
> *(ii) Hand hygiene intervention:*
> This group will receive the control intervention (health education) plus education about the potential reduction in transmission of respiratory infections to household contacts if all parties maintain proper hand hygiene, and demonstration of proper hand washing and hand antisepsis. Each household member (including the index case) will receive a uniquely labelled 100ml bottle of hypoallergenic waterless alcohol-based hand rub for individual use only, and households will be provided with one 220ml bottle of antimicrobial Ivory liquid hand soap (Proctor & Gamble, Cincinnati, OH) for each washroom and kitchen sink.
>
> *(iii) Face mask and hand hygiene intervention:*
> This group will receive the control intervention plus the hand-hygiene intervention plus education about the potential reduction in transmission of acute directly transmitted respiratory infections to household contacts if all parties wear masks, distribution of 50 (75) surgical masks for each adult (child aged 3-7) household member, and demonstration of proper face-mask wearing.

b) Randomisation plan

The pilot study will include the first two arms and a third mask-only arm (i.e. the mask intervention but not the hand hygiene intervention). For the first 100 households, we will apply an unbalanced randomisation of 2:1:1 among arms (1), (2) and (3). For the subsequent households (up to a further 100 households) we will apply an unbalanced randomisation of 8:1:1 among arms (1), (2) and (3) to allow us to extract maximum information about the transmission dynamics of influenza in the absence of non-pharmaceutical control measures. If the maximum sample size of 200 households is reached, there will be approximately 130, 35 and 35 households in arms (1), (2) and (3) respectively. Following completion of the pilot study, information derived about the characteristics of influenza transmission will be invaluable not least in validating our sample size calculation for the main study.

The main study will randomise households equally among the three arms above, using a block randomisation structure with randomly permuted block sizes of 18, 24 and 30.

We will use separate randomisation tables for subjects recruited with different criteria (as described in 4(d)) to ensure the intervention groups are balanced; this is because the QuickVue test is likely to capture subjects with on average higher viral shedding than a symptom-based criteria.

c) Medications permitted and not permitted before and/or during the trial

There are no restrictions on the use of other medications during the trial period. However the use of antivirals, antibiotics and other Western and Chinese medicine to relieve ILI symptoms during the study period will be recorded by the visiting nurse in the nurse's assessment sheet at the first and last home visits (ie. Questionnaire Q2 and Q4 respectively, appendix).

d) Procedures for monitoring subject compliance

Self-reported use of hygiene measures including mask wearing and hand washing will be recorded in the symptom diaries (Q3) by each household contact and the index case. Overall use will be reported at the final home visit, and quantities of masks, alcohol and liquid hand soap used will be measured by the visiting nurse during the final household visit.

7. Assessment of Efficacy

a) Specification of the efficacy parameter

The primary outcome measure is the secondary attack ratio (SAR) which is the proportion of household contacts with laboratory-confirmed influenza during the study period (10/7 days after recruitment in the pilot/main studies). We will preferentially use the laboratory definition of influenza rather than the clinical definition, when available.

b) Methods and timing for assessing, recording and analysing of efficacy parameter

Household contacts will be confirmed to be influenza-free at the first household visit, within 36 hours of recruitment of the index case.

In the main study, clinical influenza is defined as the presence of at least two of the following symptoms: feverishness (we will strongly encourage household members to use the supplied thermometer to assess whether a fever is ≥38°C); cough; headache; sore throat; pain in muscles or joints (following [24]). In the pilot study (as per the initial protocol), clinical influenza is defined as the presence of feverishness, or at least two of the following symptoms: cough; sore throat; nasal congestion, rhinorrhoea, or sneezing; fatigue; headache; stiffness; myalgias.

In the main study during the follow-up period of 7 days after recruitment of the index case, the index case and all household contacts will be asked to maintain a daily record of their symptoms (questionnaire Q3, appendix) and their tympanic temperature. A nurse will visit the household on three occasions during follow-up, namely 3 and 6 days after the initial home visit (day 0) with a window period of ±1

day. During each visit the nurse will collect nose swabs and throat swabs from the index case and all household contacts. These will be cryopreserved at the Department of Microbiology HKU as soon as possible as decribed in 4(n). The nose swabs and throat swabs taken during the follow-up visits will provide independent confirmation of the presence or absence of influenza virus in all household contacts, and the duration of viral shedding in the index case. In the pilot study the follow-up period will be 10 days, with visits on days 0, 3, 6 and 9.

The primary endpoint of our study will compare the SAR in each of the intervention groups with the control intervention. We will use χ^2 tests and odds ratios adjusting for potential within-household correlation, with a 5% type I error rate.

c) Diagnosis of influenza in index case

The criteria for further follow-up of the index subject and their household were described in 4(d) above. In the majority of cases, the criteria for further follow-up will be a positive result on a rapid diagnostic case. In some recruiting clinics the criteria will be presentation with at least two of the following symptoms: fever≥37.8°C (≥38°C in the pilot study); cough; headache; sore throat; pain in muscles or joints. However we will only include households in the final analyses if the index subject is laboratory confirmed to have influenza infection. This laboratory confirmation will require a positive result for influenza A or B by viral culture or standard PCR of a nose and throat swab collected from the index case at the recruitment site, and/or during the first home visit.

d) Assessment of factors which may affect the rate of influenza transmission

During the first household visit, a responsible adult (usually the household head or a parent) will be asked to provide an overview of the composition of the household, and details on past illness history and influenza vaccinations (Q2, appendix). At the final household visit, the nurse will collect information (questionnaire Q4, appendix) on the overall self/proxy-reported compliance with the intervention, and on any medication taken during the follow-up period, by asking household members and also by personally checking how many masks remain unused, or how much soap or alcohol is left in the bottles and dispensers.

8. Assessment of Safety

a) Specification of safety parameters

There are no safety parameters in this trial.

9. Statistical Analysis

a) Statistical methods to be employed

The characteristics of households, index cases and household contacts in the three intervention groups will be compared and assessed for similarity with χ^2 tests,

adjusting the comparison of household contacts for potential within-household correlation. In the primary analyses, households will only be included if the index case has laboratory-confirmed influenza infection – all other households will be dropped from the primary analysis. Furthermore, in the main study households will be dropped from the primary analysis if any of the household contacts are found to have laboratory-confirmed influenza infection at baseline although this will not be incorporated in analyses for the pilot study as per the original protocol. Therefore our results will not be biased by the potentially different transmission dynamics of other respiratory diseases compared to influenza A or B, or by the potential for more than one index case in a household when the interventions are applied.

The primary endpoint of our study will compare the SAR in each of the intervention groups with the placebo intervention (1). We will use χ^2 tests and odds ratios adjusting for potential within-household correlation [25], with a 5% type I error rate.

We will investigate the efficacy of the interventions on the SAR in multivariable logistic regression models with a generalized estimating equations approach to allow for potential within-household correlation [26]. Analysis will first be performed including only the effects of masks and hand hygiene. Further analyses will allow for the effects of potential confounders on the SAR. Confounders of the SAR that we will assess for each household contact include the age, gender, smoking status, chronic disease status, prior vaccination status, and additionally the age and gender of the corresponding index case. We will investigate the intervention effect in age/gender subgroups, although the statistical power for these analyses is unlikely to be high. We will further investigate the intervention effect in households where the intervention was applied sooner (with 36 hours) after symptom onset in the index case.

We will further investigate the intervention effects for influenza A and influenza B separately, although with likely lower incidence the statistical power for the latter may be low.

We will also assess the adherence of the index case and household contacts to the interventions, and conduct as-treated analyses of the primary outcome measure. We will conduct sensitivity analyses excluding households where the index case was prescribed antiviral medication, since onward transmission may be less likely in this scenario.

b) Planned secondary analyses

In further analyses, we will investigate the effect of the interventions on secondary outcomes listed below, and further adjust for the effect of possible confounders in multivariable logistic and proportional hazards regression models where appropriate.

1. The proportion of household contacts with clinical influenza, adjusting for the potential within-household correlation.
2. The proportion of households with one or more secondary case of influenza (laboratory definition used in preference to clinical definition where available).
3. The proportion of households with one or more secondary case of clinical influenza.

4. The time from intervention to first symptoms of clinical influenza among household contacts.

We will investigate the predictors of influenza infection and the factors affecting duration of symptoms. We will further examine the effect of environmental and lifestyle factors, and measures of risk perception on the disease course and onward transmission using regression models. We will examine the factors affecting adherence to interventions using regression models.

We will develop and apply novel modelling approaches to the analysis of the household infection data to estimate specific transmission parameters, building on previous research [27].

We will investigate the performance of the QuickVue Influenza A+B rapid diagnostic test (and the HX Diagnostics Influenza A+B test) by comparison with the gold standard of laboratory confirmed influenza by viral culture or PCR. We will further investigate the factors potentially affecting rapid diagnostic test performance, including age, gender, and time since symptom onset.

If funding is available, we will conduct further laboratory tests of collected samples to allow us to investigate the incidence and transmission dynamics of non-influenza respiratory viruses. Subjects' consent for these additional respiratory virus tests are provided on the current version and all previous versions of the informed consent forms.

If funding is available, we will apply quantitative PCR tests to nose and throat swabs collected from home visits, to investigate potential correlation between the degree of viral shedding and onward transmission, as well as the degree of viral shedding in any resulting secondary cases.

Finally, if funding is available, we will sequence the genome of influenza viruses detected in index cases and secondary cases to investigate genetic variability in the virus as well as the evolution rate between successive cases (and indeed whether secondary cases were truly infected by their corresponding household index, or from some other source).

c) Planned sample size

Pilot study: A simple calculation of the SAR is given by dividing the number of household contacts with influenza by the total number of household contacts. To estimate the anticipated SAR of 0.241 to within ±5% would require 283 household contacts. Given an average household size of 3.8 (i.e. 2.8 contacts per household) we would require at least 101 households in the placebo group; we propose to recruit 130 households in this arm to allow for some households being lost to follow-up. To test the randomization and intervention procedures we will recruit a further 35 households to the second and third study arms (masks and hand-hygiene). This will also enable a preliminary estimate of the efficacy of hand hygiene and masks although the power of the pilot study will be low to detect small or medium effect sizes with statistical significance. More importantly, the pilot study will allow an idea of the feasibility of these interventions, and coherence to them.

Main study: For the sample size calculation we require an estimate of the anticipated SAR (*P*), the degree of within-household correlation (*ρ*) in the SAR, the relative risk (*r*) that we would like to detect, and the relevant critical values of the standard normal distribution Z for a specified power (1-β) and type I error rate (α). For average household size *m*, the required number of individuals *n* in each intervention arm is given approximately by

$$n = \frac{\left(Z_{1-\alpha/2} + Z_{\beta}\right)^2 \left[P(1-P) + rP(1-rP)\right]\left[1+(m-1)\rho\right]}{(P-rP)^2},$$

and thus the number of required households is given by *n*/*m* [25].

A recent study of influenza transmission in household contacts in France found a SAR of 24.1%, and a within-household correlation of *ρ*=0.29 [28]. We will assume a reduced SAR of 20% to allow for the possibility of some transmission occurring prior to randomization and the likely inclusion of some index cases without influenza. A relative risk reduction of at least 30% is generally accepted to be clinically and epidemiologically important. We note that the efficacy of masks and hand-washing were estimated to give relative risk reductions of 90% and 75% respectively during a nosocomial outbreak of SARS, and while we doubt such high efficacy in the household setting we anticipate relative risk reductions of perhaps around 30%-50%, although there is no literature to guide us on such estimates (and hence the need for this trial!). The average household size in Hong Kong excluding houses with single or double occupancy is 3.8 (source: Hong Kong Thematic Household Survey 2002), therefore the average number of household contacts per index case would be *m*=2.8.

We would like to have at least 80% power to detect a 30% reduction (i.e. *r*=0.7) in the relative risk between intervention 2 (or intervention 3) and intervention 1 (anticipated *P*=0.2), with a 5% type I error rate. Using the formula given above we calculate that we would require the randomization of 840 household contacts into each arm of the study. Allowing for a 25% dropout rate following randomization, we would require the randomization of 840 household contacts into each study arm corresponding to a total study requirement of 2,520 household contacts in 900 households. Thus we will recruit a total of 3,420 individuals including 900 index cases with positive results on the QuickVue rapid diagnostic test, and 2,520 household contacts. The specified sample size would also have high statistical power to detect larger relative risk reductions if the observed secondary attack ratio were lower (Table 1).

Table 1: Power of proposed study (n= 840 in each arm after allowing for a 25% dropout) to detect statistically significant intervention effects of various sizes and for various secondary attack ratios, with 5% type I error.

Intervention effect (as a relative risk reduction)	Secondary attack ratio 0.25	0.20	0.15	0.10
20%	0.51	0.41	0.31	0.21
30%	0.86	0.76	0.61	0.43
40%	0.99	0.95	0.87	0.69
50%	>0.99	>0.99	0.98	0.89

To achieve this sample size, we would need to recruit approximately 6,000 suspected influenza cases and follow-up those who meet the specific criteria (in the majority of recruited subjects this would be a positive result on the QuickVue Influenza A+B rapid diagnostic test), and our reasoning is as follows. During the peak season we would conservatively anticipate that 50% of subjects with ILI symptoms, as we have defined these above, are infected with influenza rather than another virus (3,000 of the 6,000 tested). Recent international oseltamivir trials found that during the influenza peak season the proportion of subjects with influenza-like-illness who had confirmed influenza was 60% [29] and 66% [30]. Allowing for a sensitivity of 79% for the QuickVue test, only 2,370 index cases would be correctly identified (the remainder would be misclassified as false negatives). Given the estimated specificity of the QuickVue test of 92%, testing the 3,000 non-influenza index cases would result in misclassification of 240 subjects without influenza (false positives), and their households would also be visited. While the specificity of the symptom-based definition in 4(d) is likely to be lower, the sensitivity may be higher and with symptom-based recruitment only occurring in a small number of sites the approximate calculation above is appropriate. Thus we anticipate that we would need to recruit approximately 6,000 index cases with ILI symptoms and apply the specific criteria (typically the rapid diagnostic test), of who 1,477 (2,370+240) would meet our criteria and be subject to randomization and follow-up. Given the intention-to-treat approach, randomization and follow-up of some non-influenza cases is an expected consequence of the speed required by this study.

d) The level of significance to be used

We will use a significance level of α=0.05.

e) Criteria for the termination of the trial

Given the short duration of the trial, we do not plan to conduct any interim analyses or specify any early-stopping rules.

f) Procedure for accounting for any missing, unused and spurious data

The laboratory definition of influenza will be preferentially used as the primary outcome measure, but when this is unavailable we will use the clinical definition. In the pilot study, index cases and household contacts with missing data on important predictors will be excluded from analyses. In the main study, we will use multiple imputation [31] with 10 imputed datasets to replace missing values on outcome and predictor variables. If 10 imputed datasets are not sufficient to ensure stability of estimates we will use 20 imputed datasets. Multiple imputation makes maximum use of available data and maximises statistical power while requiring less strict theoretical assumptions than to a complete case analysis, or single imputation of mean values. We note that this is now one of the preferred (and standard) methods for analysing clinical trials data [32].

g) Procedures for reporting any deviations from the original statistical plan

Any deviations from the original statistical plan will be described and justified in the final report.

h) Selection of subjects to be included in the analyses

We will follow an intention-to-treat approach in the analyses. Households without a laboratory confirmed index case, and additionally in the main study those households where a household contact is laboratory confirmed to have influenza infection at baseline, will be excluded from the primary analysis.

10. Direct Access to Source Data

We will permit direct access to source data and documents for the purposes of trial-related monitoring, audits, IRB/IEC review and regulatory inspections. As required by the NIH/CDC funding agency, the *anonymised* individual participant data will be made publicly available after publication of our results in peer-reviewed journals and no later than 24 months after the conclusion of our study.

11. Ethics

An important ethical consideration is that households randomised to the control intervention might be considered to have less benefit from the trial than those assigned to the mask or hand washing interventions. However we note that there is little evidence that masks or hand washing can reduce influenza transmission, whereas this study will provide that evidence. Further, the participation of a control arm is essential to allow estimation of the effect of the interventions, given the lack of local-specific data on the SAR in typical circumstances. Thus we believe that those in the control arm, as the whole of society, will still benefit indirectly from this research.

12. Data Handling and Record Keeping

Socio-demographic and epidemiological data; confidential patient details (name address) will be collected from all subjects via the four questionnaires included in the appendix. All data will be anonymized when entered into an electronic database (double entry) and stored in the Department of Community Medicine, HKU. Original identities will be kept in a separate file accessible only to the trial manager. Original documents will be destroyed at the conclusion of the pilot study.

13. Financing and Insurance

This study is financed by a grant from the Centers for Disease Control and Prevention (Appendix A).

14. Publication Policy

The results will be published in international peer-reviewed journals.

15. References

1. Larson EL. APIC guideline for handwashing and hand antisepsis in health care settings. *Am J Infect Control.* 1995;23(4):251-269.
2. World Health Organization (WHO) Writing Group. Nonpharmaceutical Interventions for Pandemic Influenza, International Measures. *Emerg Infect Dis.* 2006;12(1):81-87.
3. World Health Organization (WHO) Writing Group. Nonpharmaceutical Interventions for Pandemic Influenza, National and Community Measures. *Emerg Infect Dis.* 2006;12(1):88-94.
4. Stohr K, Esveld M. Public health. Will vaccines be available for the next influenza pandemic? *Science.* 2004;306(5705):2195-2196.
5. Ferguson NM, Cummings DA, Fraser C, Cajka JC, Cooley PC, Burke DS. Strategies for mitigating an influenza pandemic. *Nature.* Apr 26 2006.
6. Germann TC, Kadau K, Longini IM, Jr., Macken CA. Mitigation strategies for pandemic influenza in the United States. *Proc Natl Acad Sci U S A.* 2006;103(15):5935-5940.
7. Wu JT, Riley S, Fraser C, Leung GM. Reducing the impact of the next influenza pandemic using household-based public health interventions. *PLoS Medicine.* 2006;3(9):e361.
8. Wong CM, Chan KP, Hedley AJ, Peiris JS. Influenza-associated mortality in Hong Kong. *Clin Infect Dis.* 2004;39(11):1611-1617.
9. Thompson WW, Shay DK, Weintraub E, et al. Mortality associated with influenza and respiratory syncytial virus in the United States. *JAMA.* 2003;289(2):179-186.
10. Barker J, Stevens D, Bloomfield SF. Spread and prevention of some common viral infections in community facilities and domestic homes. *J Appl Microbiol.* 2001;91(1):7-21.
11. Jefferson T, Deeks JJ, Demicheli V, Rivetti D, Rudin M. Amantadine and rimantadine for preventing and treating influenza A in adults.[update of Cochrane Database Syst Rev. 2002;(3):CD001169]. *Cochrane Database of Systematic Reviews.* 2004(3):CD001169.
12. Jefferson T, Demicheli V, Deeks J, Rivetti D. Neuraminidase inhibitors for preventing and treating influenza in healthy adults. *Cochrane Database of Systematic Reviews.* 2000(2):CD001265.
13. Demicheli V, Rivetti D, Deeks JJ, Jefferson TO. Vaccines for preventing influenza in healthy adults.[update in Cochrane Database Syst Rev. 2001;(4):CD001269; PMID: 11687102]. *Cochrane Database of Systematic Reviews.* 2000(2):CD001269.
14. Bridges CB, Thompson WW, Meltzer MI, et al. Effectiveness and cost-benefit of influenza vaccination of healthy working adults: A randomized controlled trial. *JAMA.* 2000;284(13):1655-1663.
15. Gravenstein S, Davidson HE. Current strategies for management of influenza in the elderly population. *Clin Infect Dis.* 2002;35(6):729-737.
16. Goldmann DA. Transmission of viral respiratory infections in the home. *Pediatr Infect Dis J.* 2000;19(10 Suppl):S97-102.
17. Eccles R. International scientific forum on home hygiene. Spread of the common cold and influenza. http://www.ifh-homehygiene.org/2003/2newspage/2new05.asp. Accessed 1 November, 2005.

18. Lee GM, Salomon JA, Friedman JF, et al. Illness transmission in the home: a possible role for alcohol-based hand gels. *Pediatrics.* 2005;115(4):852-860.
19. Roberts L, Smith W, Jorm L, Patel M, Douglas RM, McGilchrist C. Effect of infection control measures on the frequency of upper respiratory infection in child care: a randomized, controlled trial. *Pediatrics.* 2000;105(4 Pt 1):738-742.
20. St Sauver J, Khurana M, Kao A, Foxman B. Hygienic practices and acute respiratory illness in family and group day care homes. *Public Health Rep.* 1998;113(6):544-551.
21. Seto WH, Tsang D, Yung RW, et al. Effectiveness of precautions against droplets and contact in prevention of nosocomial transmission of severe acute respiratory syndrome (SARS). *Lancet.* 2003;361(9368):1519-1520.
22. Lo JY. Respiratory Infections during SARS Outbreak, Hong Kong, 2003. *Emerg Infect Dis.* Nov 2005;11(11):1738-1741.
23. Uyeki TM. Influenza diagnosis and treatment in children: a review of studies on clinically useful tests and antiviral treatment for influenza. *Pediatr Infect Dis J.* 2003;22(2):164-177.
24. Monto AS, Pichichero ME, Blanckenberg SJ, et al. Zanamivir prophylaxis: an effective strategy for the prevention of influenza types A and B within households. *J Infect Dis.* 2002;186(11):1582-1588.
25. Donner A, Klar N. *Design and analysis of cluster randomization trials in health research*. London: Arnold; 2000.
26. Liang K-Y, Zeger SL. Longitudinal data analysis using generalized linear models. *Biometrika.* 1986;73(1):13-22.
27. Yang Y, Longini IM, Jr., Halloran ME. Design and evaluation of prophylactic interventions using infectious disease incidence data from close contact groups. *App Stat.* 2006;55(3):317-330.
28. Viboud C, Boelle PY, Cauchemez S, et al. Risk factors of influenza transmission in households. *Br J Gen Pract.* 2004;54(506):684-689.
29. Treanor JJ, Hayden FG, Vrooman PS, et al. Efficacy and safety of the oral neuraminidase inhibitor oseltamivir in treating acute influenza: a randomized controlled trial. US Oral Neuraminidase Study Group. *JAMA.* 2000;283(8):1016-1024.
30. Hayden FG, Belshe R, Villanueva C, et al. Management of influenza in households: a prospective, randomized comparison of oseltamivir treatment with or without postexposure prophylaxis. *J Infect Dis.* 2004;189(3):440-449.
31. Schafer JL. Multiple imputation: a primer. *Stat Methods Med Res.* Mar 1999;8(1):3-15.
32. Molenberghs G, Kenward MG. *Missing data in clinical studies*. Hoboken: John Wiley & Sons Inc.; 2007.
